# Research at Great Ormond Street

**Published:** 1924-12

**Authors:** 


### RESEARCH AT GREAT ORMOND STREET.

There have recently been opened, at the Hospital for Sick Children in Great Ormond Street, a new pathological and a new biochemical laboratory. In the Pathological Laboratory investigations are being made into the causes of summer diarrhœa and rickets in children. In addition, experiments are being made as to the value of the complement fixation test in tuberculosis, and as to the precipitation of the blood serum of congenital syphilitics in relation to the findings of the Wassermann test and the clinical condition of the patients.

### Biochemical Investigations.

In the Biochemical Laboratory are to be seen, under a microscope, some specimens of the blood of the "man who turned black," who lately figured in the newspapers, who, it was found, was suffering from the effects of the presence of silver nitrate in the glands. The experiments being made are directed to discovering the nature of the crystals in the blood which have been proved to be neither cholesterol esters nor calcium soaps. Not the least important experiments relate to insulin. It has been definitely established that, up to the present, the only satisfactory method of giving it is by injection, not by the mouth nor by inunction. An instrument has been devised for estimating the respiratory quotient, in diabetics, less the carbon dioxide present; while another instrument, "The Gallows," is used for finding the amount of fibrin in the blood.

